# Parents’ and Teachers’ Views of Food Environments and Policies in Indian Private Secondary Schools

**DOI:** 10.3390/ijerph15071532

**Published:** 2018-07-19

**Authors:** Neha Rathi, Lynn Riddell, Anthony Worsley

**Affiliations:** Institute for Physical Activity and Nutrition, School of Exercise and Nutrition Sciences, Deakin University, Geelong, VIC 3220, Australia; lynn.riddell@deakin.edu.au (L.R.); anthony.worsley@deakin.edu.au (A.W.)

**Keywords:** India, school, food environment, food policies, teachers, parents

## Abstract

School food environments and policies can play a pivotal role in inculcating healthy food habits among young people. This cross-sectional survey explored teachers’ and parents’ views of the role of school food environments and policies in promoting healthy food consumption among Indian adolescents. Thirty-two teachers and 280 parents from five private, English-speaking, secondary schools in Kolkata, India took part in a short questionnaire survey which included closed and open answer questions. Descriptive and chi-square analyses were performed to compare the responses of parents and teachers. Thematic data analysis underpinned by Template Analysis Technique was employed to examine the qualitative responses. The easy availability and accessibility of energy-dense, nutrient-poor foods, the limited availability of nutritious foods, the absence of written food policies, and inflated prices of nutritious foods were reported as problems in the Indian school food environment. However, the respondents also noted that schools restricted the sale of sugar-sweetened beverages and adopted hygienic food practices. Novel ideas for creating healthy school food environments and effective school canteen policies were also captured during the survey. These findings point to the need to create effective school food policies in Indian secondary schools to help adolescents eat healthily at school. Future research is required to test the feasibility of the implementation of school food policies.

## 1. Introduction

Over the last decade there has been a growing concern regarding the increasing prevalence of overweight and obesity among Indian adolescents [1,2] which can increase the risk of developing nutrition-related chronic degenerative diseases, such as heart disease, type 2 diabetes, cancers, and pulmonary disease [3]. This burden of malnutrition poses serious threats to the health and quality of life of ‘future generations’ of adults and is a major drain on the public health resources of the country [1]. The delivery of healthy school food services supported by effective food policies could be one of the solutions for dealing with this emerging health burden [4].

The school food environment serves as a potential target for improving the health and nutritional status of young people [5] for two reasons. First, schools provide an unparalleled opportunity to reach a large segment of young people, their families, and the wider community across different cultural and socio-demographic settings [6]. Second, young people consume approximately one-third of their daily meals at school [7,8]. In general, schools can adopt several specific actions to improve the food habits of young people. These include the greater availability and accessibility of fruits and vegetables, the application of consistent nutrient-based standards on the foods and meals supplied in schools, monetary incentives for healthy food choices at point-of-purchase, and skills-oriented nutrition education for students, teachers, and catering staff [4].

Given the contribution schools make to adolescent food habits, it is disappointing to note that majority of school food environments in both economically developed [9,10] and developing countries [11,12] foster unhealthy food habits in young people. There is ample evidence from western countries to suggest that school canteens (tuckshops or cafeterias) predominantly supply a wide variety of energy-dense, nutrient-poor foods (e.g., French fries, confectionery) and sugar-sweetened beverages [13,14,15]. This in turn, further triggers consumption of these unhealthy food products in young people [16]. In contrast, there is limited exposure to nutritious foods in many school canteens, thus, inhibiting students from making healthy food choices [12,17]. These unhealthy canteen practices may contribute to the global burden of obesity [16].

Despite the plethora of international evidence, scant scientific information is available about the Indian school food environment. However, a recent qualitative investigation [18] that explored the views of adolescents, their parents, teachers, and school principals on food environments and policies in Indian secondary schools replicate international findings. It found that Indian schools predominantly lack written food and health policies [18], a finding also confirmed in previous studies [19,20]. This may be one reason behind the unhealthy food services provided in Indian schools [18,21]. The interviewees also reported that schools practised unhygienic canteen practices and sold certain food products at inflated prices [18]. Similar negative criticism of school canteens has been reported in a number of previous investigations [22,23,24,25]. In addition, the inconsistency between the unhealthy school food supply and healthy eating communications disseminated in nutrition lessons also emerged as a problem during this qualitative inquiry [18], a criticism often reported in the literature [26,27,28].

Considering the relative scarcity of information about the Indian school food environment as well as the limited generalisability of qualitative findings, a larger, descriptive study was warranted. Therefore, the present study aimed to examine the perspectives of teachers and parents about the current school food environments and their views of possible future healthy school food environments and policies. The views of parents and teachers are important because the successful implementation of school food policies involves the cooperation of these two groups of stakeholders [29,30].

## 2. Materials and Methods

### 2.1. Research Design and Sampling

This study was carried out as a part of a detailed cross-sectional survey which examined the Indian secondary school food and nutrition landscape. The survey was conducted between August and November 2016. Parents of year 9 students and Biology and Home Science teachers from five private English-speaking secondary schools (three co-educational schools, one-single-sex boys’ school, and one-single sex girls’ school) in Kolkata, India were invited to participate in the survey. The schools were selected through convenience sampling from five different geographic locations (i.e., east, west, north, south, and central) in Kolkata city. Private schools were selected because there is a greater prevalence of overweight and obesity among private school students than public school students [21,31]. Moreover, private school students make up about 40 percent of Indian secondary students [32]. Methodological details for this survey have also been reported previously [33].

Biology and Home Science teachers responsible for teaching nutrition to year 9 students were recruited because they were expected to be well-informed about the school canteen and school food policies. Parents of year 9 students were chosen as potential informants because they are recognised as the primary food gatekeepers of their adolescents. Past research indicates that parents may play a key role in influencing adolescents’ food habits [34,35,36,37]. Moreover, they are generally well aware of their adolescent’s school food environment and policies [18].

### 2.2. Survey Instrument

An anonymous, self-reported School Food Landscape Questionnaire (SFLQ) was specifically designed for examining the food and nutrition situation in Indian schools. This 16-page instrument printed in English included 115 close-ended questions and eight open-ended questions about food and nutrition education, food skills development, school food services and policies, adolescent food habits, human values, and socio-demographic characteristics of the sample.

This paper exclusively focuses on items related to school food services and policies. Fourteen statements were used to obtain the stakeholders’ views of the current school food environment and policies. In addition, a series of statements (*n* = 18) were used to elicit respondents’ expectations of potential future healthy school food environments and policies.

All these 32 close-ended questions employed five-point Likert response scales ranging from “strongly disagree” (coded as 1), “disagree” (2), “neutral” (3), “agree” (4), to “strongly agree” (5). In addition, one open-ended question was included to capture the respondents’ views of the ideal school food policy (‘*What is your view of an ideal school canteen policy*?’). These survey items were informed by findings from a recent qualitative investigation [18].

### 2.3. Procedure

The questionnaire was pilot tested for readability and clarity with a convenience sample of nine teachers and 21 parents recruited from a private secondary school in Kolkata. This participating school was not a part of the main survey. Based on these respondents’ feedback, minor modifications were made to the questionnaire. The pilot data were not merged with the main data set.

Prior to administration of the survey, the principals of the five schools were informed about the survey procedures. They were required to grant permission by completing and returning the Organisational Consent Form to the senior author (NR). All the eligible teachers (*n* = 35) were presented with a Plain Language Statement and Consent Form, a questionnaire and an envelope on school grounds. The school authorities attached this recruitment pack to all year 9 students’ diaries in order to request parental participation (*n* = 309).

The respondents were asked to complete and return the questionnaires and consent forms in sealed envelopes to the school authorities within seven days. A reminder was sent to them if they failed to return the completed questionnaires within the specified time period. Subsequently, three weeks after the administration of the survey, the senior author (NR) collected all the sealed envelopes from the school authorities. The respondents did not receive any gifts or material incentives for participating in the survey. Deakin University’s Health Ethics Advisory Group (HEAG-H 127_2016) provided ethical approval for this research investigation.

In total, 312 respondents (32 teachers; 280 parents) completed the survey, an overall response rate of 90.7%. The response rate for teachers and parents was 91.4% and 90.6% respectively. An adequate statistical power (83%) was achieved for the study with an effect size of 0.2 (Cohen’s w used in chi-square tests), at a significance level of 0.01.

### 2.4. Data Analysis

Examination of the quantitative data was undertaken using the Statistical Package for Social Sciences (SPSS) version 22.0 (IBM Corporation, Armonk, NY, USA). The distributions of the variables were examined. For analysis purposes, the five point Likert scales were collapsed to form three point scales, collapsing ‘strongly agree’ and ‘agree’ to form one category, ‘strongly disagree’ and ‘disagree’ to form another, while leaving ‘neutral’ as a separate category. Descriptive statistics and two-by-two contingency tables were performed.

A manual qualitative data analysis method underpinned by the Template Analysis technique [38] was used on the open-ended question. This thematic technique involved the development of codes (in a ‘template’) indicative of the themes specified in the textual data. A hierarchical template was developed which depicted the associations between themes. The lead author (NR) initially coded and analysed the data. Subsequently, inter-rater reliability [39] was verified by two professionals (one home economist and one nutritionist) who independently analysed a subset of 30 questionnaires. Any differences were resolved by discussion until a consensus was reached that the established themes were representative of the data [40]. Consequently, the main themes and relevant quotations based on the reports were produced.

## 3. Results

### 3.1. Socio-Demographic Characteristics of the Sample

The mean age of the sample was 41.91 years (SD = 4.46 years; range 25–55 years). About two-thirds (68.6%, Table 1) of the sample were women. The majority of the respondents (89.7%) had a university degree.

### 3.2. Quantitative Findings

Around three-fifths of the participants agreed on the widespread availability of unhealthy foods in the school canteen (59.9%, Table 2). However, about four-fifths agreed that carbonated beverages were not supplied in school canteens (80.8%). Approximately half of the respondents (53.8%) agreed with the statement that there was limited availability of healthy food items in the school canteen. Nearly half of the sample agreed that canteen menus lacked variety (47.4%) and food items sold in the canteen were expensive (44.9%). Around four-fifths of the respondents agreed that canteens only sell vegetarian products (82.1%). Just over half of the respondents agreed that good quality food ingredients were used in preparing canteen food (55.4%) and few agreed that the canteen was unhygienic (14.7%). Only two-fifths agreed that there was a written school food policy and few participants agreed that parents (16.7%), adolescents (11.2%), and teachers (23.4%) were consulted during the preparation of canteen menus.

Overall, the contingency table analyses revealed close similarities between teachers and parents in their perceptions of the present school food services and policies in secondary schools. However, a significantly higher proportion of parents (62.5%) than teachers (37.5%) agreed with the statement ‘A wide variety of unhealthy foods (e.g., French fries) is available in the school canteen’ (*p* < 0.01, Table 2). Similarly, more parents than teachers agreed with the statements that parents and adolescents were consulted while planning the canteen menu (*p* < 0.01).

Most of the respondents (92.9%) acknowledged the need for a canteen in every school (Table 3). Further, the majority of the sample agreed with the statements that school canteens should not sell unhealthy foods (89.7%), should promote the sale of healthy foods at a reasonable price (96.2%), and that the food should be palatable (96.5%). There was support for maintaining hygiene and sanitation in the school food environment (98.1%). The provision of safe drinking water on school premises attracted the support of almost all the respondents (99.0%). Nearly, three-quarters of the sample agreed with the statement that canteens should sell both vegetarian and non-vegetarian food items (71.8%). Most respondents agreed that school food services should complement the food and nutrition curriculum (94.2%). Around four-fifths of the respondents agreed that adolescents should consume home-prepared lunches at school on a regular basis (80.1%) and canteen food should be consumed occasionally (76.9%).

In general, there were no significant differences between parents and teachers in their perceived strategies of improving the quality of secondary school food services. However, more parents (82.1%) than teachers (62.5%) agreed that adolescents should consume home-prepared lunches on a regular basis (*p* < 0.01; Table 3).

### 3.3. Qualitative Findings

The respondents identified a number of themes associated with an ideal school food policy. These included the provision of tasty, healthy foods at reasonable prices; restricted availability of unhealthy foods and beverages; consistency between nutrition lessons and school food services; maintenance of hygiene and sanitation in canteens; routine canteen checks; and involvement of all the key stakeholders in policy development. Many respondents (92.9%, Table 4) suggested that an ideal food policy should be one which allows the school canteen to offer an adequate number of healthy food options to students (All the percentages specified in this qualitative section represent the percentages of respondents reporting particular themes). In addition, the canteen policy should limit the availability of unhealthy foods like sugar-sweetened beverages and fast food.

*The school canteen should have written policy and they should serve healthy foods like salad, fresh fruits that are good for the health of adolescents.*

(Parent 63)

*Junk food should be completely banned!*

(Teacher 12)

A common recommendation (made by 95.2% of respondents) was that classroom teaching should complement the school food services. One parent described it as:

*The school canteen should sell only those foods which the children have been taught to have daily in the home science books.*

(Parent 20)

The cost, palatability, and appearance of canteen foods were described as essential components of an effective school food policy. Almost four-fifths of the respondents (78.8%) recommended that healthy foods served to students should be economical, attractive, and tasty.

*Make the healthy food look attractive and tasty.*

(Teacher 18)

*Food should be fresh and healthy. It should be reasonably priced.*

(Parent 141)

Hygiene and sanitation were cited as important attributes of an ideal canteen policy. Many teachers and parents stated that school canteens should maintain a hygienic, clean environment. They also noted that canteen personnel should wear neat and clean uniforms as well as adopt hygienic food handling practices.

*A clean and a hygienic environment is a must!*

(Parent 179)

*The person serving food should be well-dressed with gloves.*

(Teacher 4)

Almost all the respondents (99%) emphasised the provision of safe drinking water in the canteen. The use of fresh food ingredients in preparing canteen foods was also specified as an integral component of an ideal canteen policy.

*The school canteen should sell fresh and healthy food products along with the supply of safe and clean drinking water*……

(Teacher 7)

*Safe drinking water should be provided.*

(Parent 139)

*See good quality food ingredients must be used in food preparation as children are having these stuff!*

(Parent 261)

There was support (24.7%) for the conduct of routine audits/inspections to ensure that the canteen staff comply with canteen policy guidelines. This surveillance could minimise the violation of rules and regulations as specified by the respondents. In addition, in-house training of the canteen staff in promoting the sale of healthy foods was viewed as a vital healthy eating strategy.

*The management should conduct frequent surprise checks over the canteen foods.*

(Parent 12)

*These canteen vendors should be trained to prepare and sell healthy stuff!*

(Teacher 32)

*Always check the food, coming outside of the school or derived by other. Canteen staff should be professional i.e., having food sense. Canteen should be under surveillance.*

(Parent 224)

The need for consulting parents, teachers and adolescents in developing an ideal school canteen policy also emerged as a key theme (80.1%). One parent described it as follows:

*The school canteen should consider the opinions of parents, teachers to frame the school canteen menu. They should speak with the children to make them aware of all the healthy food being kept in the school canteen.*

(Parent 60)

## 4. Discussion

This investigation set out to develop a better understanding of the private Indian secondary school food environments and policies, from the perspective of teachers and parents, who are acknowledged as key stakeholders in education settings [41]. These crucial stakeholders expressed both positive and negative views about the current school food environments and policies. They also advanced innovative ideas for creating healthy school food environments and effective school canteen policies.

The easy availability and accessibility of energy-dense, nutrient-poor foods (e.g., French fries in the Indian school canteens) invited strong criticism from several stakeholders. Similar criticism of school canteens has been reported previously [10,11,18]. For example, Brazilian and Malaysian school canteens have heavily promoted non-nutritious snacks among pupils [11,12]. Findings from industrialised nations like the USA [10] and Canada [42] also reflect similar unhealthy school environments. These environments are frequently associated with adolescents’ increased consumption of sugar-sweetened beverages and energy-dense, nutrient-poor foods (such as pizza, chips, and candies) [7,9]. Indeed, Indian adolescents demonstrate unhealthy eating habits [43,44]. Overconsumption of unhealthy foods and beverages is viewed as an important contributor to adolescent obesity [45].

In line with previous findings [18], the stakeholders expressed dissatisfaction over the limited supply of healthy food items in the school canteen. This poor availability of healthy foods interferes with adolescents’ ability to consume these foods [46]. Moreover, young people lack the maturity and cognitive ability to make the healthiest food choices, particularly in a contemporary society where they are heavily exposed to sophisticated food marketing [46].

In agreement with the principals’ views in our recent qualitative investigation [18], several respondents (80.8%) also noted that carbonated beverages (e.g., Coke) were not supplied in their schools’ canteens. This ban seems to be a positive step towards the reduction of obesity in young people. A number of economically developed and developing countries like France, England, Scotland, Portugal [47], South Korea [48], Thailand [47], and Sri Lanka [49] have also imposed restrictions on the supply of sugar-sweetened beverages in the school premises to address adolescent obesity. Nonetheless, scant published evidence is available on the effectiveness of these policies [47]. Further, the availability of these beverages in the school neighbourhood needs to be investigated.

Several respondents reported that only vegetarian food items were sold in the school canteen, a finding also observed in the qualitative investigation [18]. This practice of Indian schools is influenced by different religious philosophies including Hinduism, Jainism, and Buddhism [50]. This vegetarian practice may restrict the intakes of important sources of bioavailable iron and zinc among other nutrients [51]. Interestingly, most of the vegetarian products (e.g., French fries, samosa) available in the school canteen were high in energy and low in nutrients. Quite often, these food products were deep-fried in trans fats and saturated fats [52]. Some participants indicated the need for introducing non-vegetarian products in the school canteen. The introduction of iron-rich animal-based products [53] could help to improve dietary intakes of micronutrients such as iron and zinc. Considering the dominance of Hindu respondents, the probability of purchasing and consuming these animal products remains questionable.

In contrast to previous qualitative findings [18], only a small proportion of the sample observed that schools exhibited unhygienic canteen practices. A number of deficiencies related to the use and change of hand-gloves, cleanliness of work stations and hand-washing have resulted in unhygienic canteens [54,55]. The poor hygiene procedures employed by canteen personnel have been recognised as significant contributors of foodborne infections in young people [23,24]. Probably, because of the danger of such foodborne infections, the respondents reported the need for safe drinking water and a hygienic canteen, initiatives endorsed by the Focus Resources on Effective School Health (FRESH) framework [56].

Most respondents indicated that school canteens supplied nutritious foods at inflated prices. Comparable views have been reported in both local [18] and overseas investigations [57]. School canteens are primarily profit-oriented; they mostly rely on the revenue generated through the sale of foods high in fat or sugar [57]. Therefore, the respondents proposed that schools should supply tasty nutritious foods at economical prices. Studies of Nordic [58] and Australian adolescents [59] have also suggested that increased accessibility of tasty nutritious meals and snacks at a low cost could help them to make healthier food choices. Taste is an important determinant of food choice [60]. It has been claimed that adolescents often prefer to consume fast food over healthy foods primarily because of their taste preference [12,49]. This highlights the need for enhancing the palatability of healthy food items to enhance their consumption among students.

Some participants stated that parents, adolescents, and teachers tended not to be consulted during menu planning, a view consistently expressed by the interviewees in the qualitative study [18]. In contrast, Dutch [61], Portuguese [62], and Chinese [63] schools encourage the participation of all the stakeholders in school operations, a crucial initiative in improving the healthiness of the school canteens [29,30,64,65]

Only two-fifths of the respondents claimed that schools had a written food policy. Previous local studies have also shown comparable findings [18,19,20]. Perhaps, the lack of an Indian or State Government mandate is responsible for this poor state of the Indian school food environment. Unlike India, Government agencies in Sri Lanka [49] and South Korea [48] have developed canteen policies for their respective schools. Government mandated school food policies may not be popular. In the UK, many staff and students did not support government policies that restricted certain foods and minimised personal preferences [64]. Similarly, Malaysian [12] and Sri Lankan [49] schools also reported opposition to the imposition of food policies. Such opposition can generally be avoided through careful consultation with all the school stakeholders, an approach recommended by a number of public health experts [29,30].

Undoubtedly, effective school food policies are essential for providing healthy foods at school, but proper training of canteen staff is also a primary prerequisite for the successful implementation of these school food policies. For example, the New South Wales ‘Fresh Tastes @ School’ program enabled the canteen staff to undertake special TAFE (Technical and Further Education) courses in nutrition and business management [66]. This program was successful in reducing the purchase of unhealthy foods in school cafeterias [66].

Ideally, a school food policy should involve a series of actions that take into account all the characteristics of the school population [4]. Therefore, actions should be targeted at creating a healthy learning environment through repeated and sustained exposure to nutritious foods and consistent nutrient-based standards for the foods and drinks available in schools [4].

The limitations of this survey should be acknowledged while interpreting the findings. First, because of the cross-sectional design of the survey, causal associations between variables cannot be made. Second, the use of convenience sampling in the selection of five private schools could have limited the generalisability of the findings. Because of logistic reasons, random sampling could not be implemented in the present scenario. Third, the survey findings were limited to private schools in Kolkata and this might have further minimised the representativeness of the sample. Moreover, the sample was representative of an urban middle class Bengali culture as indicated by their educational profile. Future research should examine school food environments and policies in other urban and rural areas in India. In addition, the food and nutrition situation in public schools also needs examination. Furthermore, to gain a more comprehensive perspective, the opinions of other key stakeholders including school principals [67] and canteen personnel [68] should also be examined in future studies.

However, this study had several strengths. The present findings are unique as it is the first cross-sectional survey to explore parents’ and teachers’ perceptions of the food environment and policies in Indian school settings. A further strength was the high response rate (90.7%). The use of one open-ended question provided the opportunity to explore respondents’ views of an ideal school food policy in greater depth.

## 5. Conclusions

Almost all the parents and teachers were able to identify strategies to improve the food and nutrition environment in schools. The findings suggest that Kolkata schools are currently not providing optimal selection of a variety of palatable nutritious foods at an appropriate price. This highlights the need for developing healthy canteen policies in consultation with stakeholders and possibly mandating them in future. The respondents’ mutual interest and enthusiasm in improving the school food and nutrition situation is likely to be instrumental in informing the development of healthy school food policies in India.

## Figures and Tables

**Table 1:** Socio-demographic characteristics of the participants (*n* = 312).

	Parents % (*n*)	Teachers % (*n*)	Total % (*n*)
**Gender**			
Male	35.0 (98)	0.0 (0)	31.4 (98)
Female	65.0 (182)	100.0 (32)	68.6 (214)
**Educational Qualification**			
Secondary school (Till class 10)	1.4 (4)	0.0 (0)	1.3 (4)
Higher secondary school (Till class 12)	10.0 (28)	0.0 (0)	9.0 (28)
University qualification (e.g., B.Sc., M.Com)	88.6 (248)	100.0 (32)	89.7 (280)

**Table 2:** Respondents’ views of the current food environment and policies in Indian secondary schools (% Strongly agree *, *n* = 312).

	Parents % (*n*)	Teachers % (*n*)	Total % (*n*)	χ^2^	df	*p*-Value
**A wide variety of unhealthy foods (e.g., French fries) is available in the school canteen**	**62.5 (175)**	**37.5 (12)**	**59.9 (187)**	**9.829**	**2**	**<0.01**
Very few healthy food items (e.g., salads) are available in the school canteen	54.6 (153)	46.9 (15)	53.8 (168)	1.180	2	0.55
Fizzy drinks (e.g., Coke) are not sold in the school canteen	80.4 (225)	84.4 (27)	80.8 (252)	3.316	2	0.19
The school canteen is unhygienic	15.7 (44)	6.3 (2)	14.7 (46)	3.166	2	0.21
The school canteen only sells vegetarian food products	82.5 (231)	78.1 (25)	82.1 (256)	2.119	2	0.38
Foods supplied in the school canteen are expensive	46.8 (131)	28.1 (9)	44.9 (140)	6.375	2	0.04
The school canteen menu lacks variety	47.9 (134)	43.8 (14)	47.4 (148)	1.112	2	0.57
The school canteen used good quality ingredients in food preparation	54.3 (152)	65.6 (21)	55.4 (173)	1.582	2	0.45
Adolescents carry home-prepared packed lunch to school	76.8 (215)	68.8 (22)	76.0 (237)	1.559	2	0.46
Home-prepared packed lunch is healthier than food supplied in the school canteen	74.6 (209)	59.4 (19)	73.1 (228)	3.633	2	0.16
Schools have a written canteen policy	41.1 (115)	34.4 (11)	40.4 (126)	0.653	2	0.72
**Parents are consulted while planning the canteen menu**	**18.2 (51)**	**3.1 (1)**	**16.7 (52)**	**25.387**	**2**	**<0.01**
**Adolescents are consulted while planning the canteen menu**	**12.1 (34)**	**3.1 (1)**	**11.2 (35)**	**13.125**	**2**	**<0.01**
Teachers are consulted while planning the canteen menu	23.9 (67)	18.8 (6)	23.4 (73)	4.650	2	0.09

* Scale: 3-point scale 1 + 2 = 1 strongly disagree/disagree, 3 = 3 neutral, 4 + 5 = 5 agree/strongly agree; Measures only significant at *p* < 0.01 are highlighted in bold.

**Table 3:** Respondents’ views of strategies to improve school food services (% Strongly agree *, *n* = 312).

	Parents % (*n*)	Teachers % (*n*)	Total % (*n*)	χ^2^	df	*p*-Value
Every school should have a canteen	92.9 (260)	93.8 (30)	92.9 (290)	2.253	2	0.32
The school canteen should not sell unhealthy foods (e.g., French fries, fizzy drinks)	88.6 (248)	100.0 (32)	89.7 (280)	4.075	2	0.13
The school canteen should promote the sale of healthy foods (e.g., salads)	95.0 (266)	96.9 (31)	95.2 (297)	0.221	1	0.64
Healthy foods sold in the school canteen should be tasty	96.1 (269)	100.0 (32)	96.5 (301)	1.303	2	0.52
Healthy foods sold in the school canteen should be reasonably priced	95.7 (268)	100.0 (32)	96.2 (300)	1.426	2	0.49
Hygiene and sanitation should be maintained in the school canteen	97.9 (274)	100.0 (32)	98.1 (306)	0.699	2	0.76
Safe drinking water should be available in the school canteen	98.9 (277)	100.0 (32)	99.0 (309)	0.346	1	0.56
Both vegetarian and non-vegetarian food products should be available in the school canteen	70.4 (197)	84.4 (27)	71.8 (224)	3.094	2	0.21
Adolescent participation in the functioning of the school canteen must be encouraged	81.1 (227)	90.6 (29)	82.1 (256)	2.473	2	0.29
Parent participation in the functioning of the school canteen must be encouraged	71.1 (199)	56.3 (18)	69.6 (217)	3.596	2	0.17
Teacher participation in the functioning of the school canteen must be encouraged	80.4 (225)	65.6 (21)	78.8 (246)	5.729	2	0.06
Food sold in the school canteen should complement healthy eating messages delivered in food and nutrition classes	94.6 (265)	90.6 (29)	94.2 (294)	1.532	2	0.47
**Adolescents should consume home-prepared packed lunches on a regular basis**	**82.1 (230)**	**62.5 (20)**	**80.1 (250)**	**13.015**	**2**	**<0.01**
Canteen food should be consumed occasionally	77.5 (217)	71.9 (23)	76.9 (240)	0.525	2	0.77
A written canteen policy is essential	84.3 (236)	71.9 (23)	83.0 (259)	3.137	1	0.78
Adolescents should be consulted during the development of school canteen policy	78.6 (220)	65.6 (21)	77.2 (241)	3.733	2	0.16
Parents should be consulted during the development of school canteen policy	80.4 (225)	71.9 (23)	79.5 (248)	3.814	2	0.15
Teachers should be consulted during the development of school canteen policy	85.4 (239)	71.9 (23)	84.0 (262)	5.409	2	0.07

* Scale: 3-point scale 1 + 2 = 1 strongly disagree/disagree, 3 = 3 neutral, 4 + 5 = 5 agree/strongly agree; Measure only significant at *p* < 0.01 is highlighted in bold.

**Table 4:** Respondents’ views of an ideal school food policy (*n* = 312) *.

	Respondents % (*n*)
Theme 1: Widespread availability of healthy foods	92.9 (290)
Theme 2: Restricted availability of unhealthy foods and beverages	82.1 (256)
Theme 3: Canteen food should be attractive, tasty, and reasonably priced	78.8 (246)
Theme 4: Consistency between nutrition lessons and school food services	95.2 (297)
Theme 5: Maintenance of hygiene and sanitation in canteens	99.0 (309)
Theme 6: Routine canteen checks	24.7 (77)
Theme 7: Involvement of all the key stakeholders in policy development	80.1 (250)

* All the percentages specified in this Table represent the percentages of respondents reporting particular themes.
